# Oncogenic transformation of mammary epithelial cells by transforming growth factor beta independent of mammary stem cell regulation

**DOI:** 10.1186/1475-2867-13-74

**Published:** 2013-07-25

**Authors:** Karen A Dunphy, Jae-Hong Seo, Daniel J Kim, Amy L Roberts, Luwei Tao, James DiRenzo, Amanda L Balboni, Giovanna M Crisi, Mary J Hagen, Thiruppavai Chandrasekaran, Kelly J Gauger, Sallie Smith Schneider, D Joseph Jerry

**Affiliations:** 1 Veterinary and Animal Sciences, University of Massachusetts, Amherst, MA 01003, USA; 2 Korea University, Seoul, Korea; 3 Dartmouth Medical School, Hanover, NH 03755, USA; 4 Baystate Medical Center, Springfield, MA 01107, USA; 5 Pioneer Valley Life Sciences Institute, Springfield, MA 01107, USA

**Keywords:** Transforming growth factor beta, TGFβ, Epithelial to mesenchymal transition, EMT, Transdifferentiation

## Abstract

**Background:**

Transforming growth factor beta (TGFβ) is transiently increased in the mammary gland during involution and by radiation. While TGFβ normally has a tumour suppressor role, prolonged exposure to TGFβ can induce an oncogenic epithelial to mesenchymal transition (EMT) program in permissive cells and initiate the generation of cancer stem cells. Our objective is to mimic the transient exposure to TGFβ during involution to determine the persistent effects on premalignant mammary epithelium.

**Method:**

CDβGeo cells, a transplantable mouse mammary epithelial cell line, were treated *in vitro* for 14 days with TGFβ (5 ng/ml). The cells were passaged for an additional 14 days in media without TGFβ and then assessed for markers of EMT and transformation.

**Results:**

The 14-day exposure to TGFβ induced EMT and transdifferentiation *in vitro* that persists after withdrawal of TGFβ. TGFβ-treated cells are highly tumorigenic *in vivo*, producing invasive solid de-differentiated tumours (100%; latency 6.7 weeks) compared to control (43%; latency 32.7 weeks). Although the TGFβ-treated cells have initiated a persistent EMT program, the stem cell population was unchanged relative to the controls. The gene expression profiles of TGFβ-treated cells demonstrate de-differentiation with decreases in the expression of genes that define luminal, basal and stem cells. Additionally, the gene expression profiles demonstrate increases in markers of EMT, growth factor signalling, TGFβ2 and changes in extra cellular matrix.

**Conclusion:**

This model demonstrates full oncogenic EMT without an increase in stem cells, serving to separate EMT markers from stem cell markers.

## Background

Transforming growth factor beta (TGFβ) has paradoxical roles in breast cancer acting as both a tumour suppressor and tumour promoter [1-4]. In the normal mouse mammary epithelium, tumour resistance is achieved with TGFβ-mediated cell cycle arrest and apoptosis [5-7]. TGFβ also initiates epithelial-mesenchymal transition (EMT) [2]; whereby epithelial cells lose cell adhesions and polarity and assume a mesenchymal motile phenotype. The EMT process is transient, and cells usually revert to their former phenotype via mesenchymal-epithelial transition (MET).

Transient non-oncogenic EMT is a normal cellular program that initiates cell migration during embryogenesis to direct organ development; and, in differentiated tissues, directs wound healing, regeneration and remodelling [8]. TGFβ is normally expressed in the mammary gland and contributes to spatial distribution of the epithelial tree by regulating ductal elongation and branching [9,10]. Expression of TGFβ is increased during involution of the mammary gland following pregnancy [11], and consequently, TGFβ-mediated apoptosis and cell cycle arrest reduce epithelial content to proportions found in the non-lactating gland [12,13]. Likewise, a natural transient TGFβ-mediated EMT program is employed to remodel the mammary ductal tree during the involution process.

However, elevated levels of TGFβ during pregnancy and involution can initiate a persistent oncogenic EMT program in premalignant epithelial cells leading to tumour initiation and development of pregnancy associated breast cancer (PABC) [14]. TGFβ is also activated by radiation therapy [15,16], and may increase the metastatic behaviour of an existing cancer by promoting EMT [17]. However, TGFβ-mediated EMT is a rare event *in vitro*[18] and experiments demonstrating TGFβ induction of persistent oncogenic EMT are limited to mammary epithelial cells that have already been transformed with activated Ras oncogene [19-21] or cells that are persistently exposed to TGFβ [22]. Normal mouse and human mammary epithelial cells only undergo transient EMT in response to TGFβ *in vitro*[23,24], but revert to an epithelial phenotype via MET after TGFβ withdrawal and remain non-tumorigenic *in vivo*.

TGFβ-mediated EMT may also promote the generation of cancer stem cells [20,25,26]. Induction of EMT and a mesenchymal state increased the population of CD24^Low^ CD44^High^ stem cells in mammary carcinoma cell lines [27,28]. Because there is considerable overlap in gene expression profiles linking cells undergoing EMT with stem cells [29,30], including up-regulation of Snail, Zeb2 and down-regulation of Sfrp1 [31,32], signatures for EMT and stem cells have been difficult to separate.

The CDβGeo mouse mammary epithelial cell line is a heterogeneous population of K8+ luminal epithelial and K5+ basal cells that are enriched for progenitors [33]. This cell line was derived from COMMA-D cells and, although phenotypically heterogeneous, the cells are clonal with respect for biallelic mutations in *Trp*53 [34]. These cells form epithelial ductal trees when transplanted into cleared mouse mammary fat pads and are mildly tumorigenic [35]. In our experiments using the CDβGeo cell line, we have generated a model whereby this mouse epithelial cell line is transformed by transient TGFβ-treatment *in vitro* making it highly tumorigenic *in vivo*, yet the increased tumorgenicity did not alter the stem cell pool. The transient TGFβ-treatment stimulates an autocrine TGFβ loop supporting persistent EMT with sustained expression of Snail, but inhibition of TGFβRI only imparts partial rescue.

## Results

### Transient TGFβ exposure causes persistent transdifferentiation in CDβGeo cells

**Figure 1:**
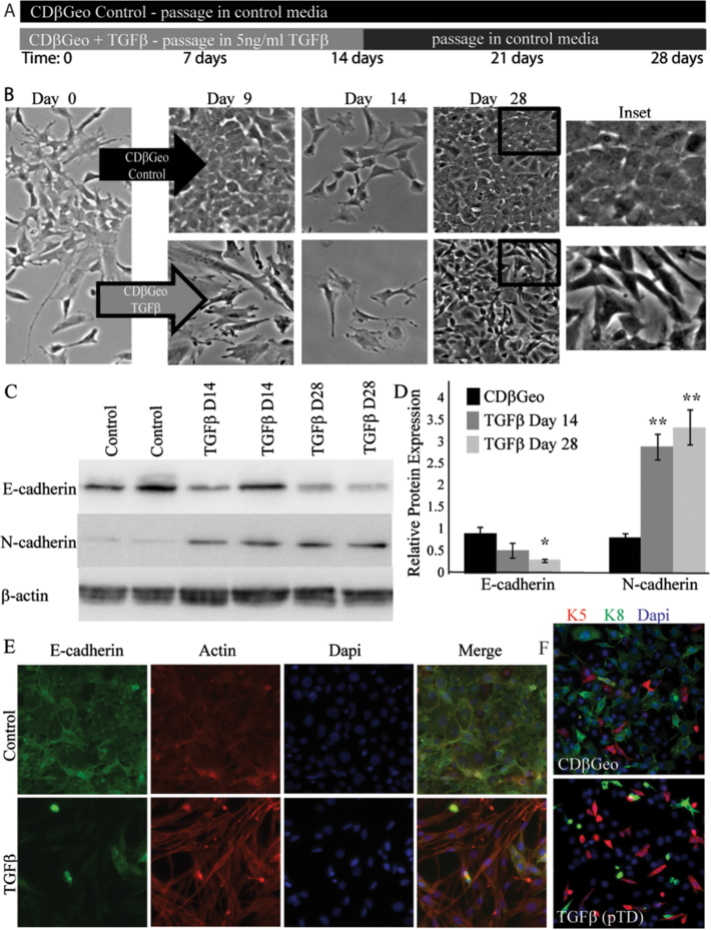
**Transient TGFβ exposure causes persistent transdifferentiation in CDβGeo cells. (A)** Description of experimental timeline – Cells were cultured and passaged in control media for 28 days (CDβGeo control) or 5 ng/ml TGFβ for 14 days followed by passage in control media for an additional 14 days. **(B)** The parental CDβGeo cells retain a cuboidal cobblestone morphology throughout the treatment period in the control media, but during TGFβ-treatment cells become senescent, spindle-like and avoid adhesion. **(C)** Western blot analysis shows down-regulation of E-cadherin and up-regulation of N-cadherin **(D)** Quantification of E-cadherin and N-cadherin protein expression, normalized to β-actin (* p < 0.05; **p < 0.01). **(E)** Immunofluorescence for E-cadherin and Actin demonstrate appropriate expression in control cells. TGFβ-treated cells have reduced E-cadherin expression and actin stress fibers. **(F)** K8 expression is reduced in TGFβ-treated cells.

CDβGeo cells were passaged for 14 days in DMEM:F12 media with solvent control or 5 ng/ml TGFβ1 to mimic mouse TGFβ exposures during involution (Figure 1A). Morphological changes in the CDβGeo cells occurred 4–5 days after TGFβ exposure. In contrast to CDβGeo cells maintained in control media which exhibit a cuboidal epithelial phenotype and attained confluence several times during the 14 day treatment period, TGFβ-treatment reduced cell growth (Additional file 1: Figure S1A) and cells exhibited a senescent phenotype (Figure 1B). After the 14 day treatment period, TGFβ was withdrawn and cells recovered and grew to confluency. With the exception of a few isolated epithelial-like patches, the TGFβ-treated cells remained spindle-shaped and did not resume the cuboidal epithelial phenotype (Figure 1B: compare day 28 and inset).

The TGFβ-treated CDβGeo cells have reduced expression of E-cadherin (p < 0.05) and significantly increased expression of N-cadherin (p < 0.01) relative to CDβGeo control cells after 14 days (Figure 1 C&D). The expression of E-cadherin is further reduced in the ensuing 14 days (day 28), even though TGFβ had been removed. Immunofluorescence on day 28 demonstrates appropriate expression of E-cadherin and actin at the cell borders of the epithelial CDβGeo control cells (Figure 1E), while few TGFβ-treated cells express any E-cadherin. In the TGFβ-treated cells, the actin filaments are arranged in longitudinal lines of stress, indicative of loss of epithelial phenotype and acquisition of a mesenchymal phenotype. In cell culture, the CDβGeo cells produce a heterogeneous cell population, including cells that express the luminal epithelial cytokeratin (K8) and a smaller percentage of cells that express the basal cytokeratin (K5) (Figure 1F). Expression of K8 is lost in the TGFβ-treated cells such that the K5 population is increased (Additional file 1: Figure S1B). These results support the conclusion that the CDβGeo cells have undergone persistent transdifferentation. These phenotypic changes were reversible after prolonged culture (>10 passages) with E-cadherin levels being restored. Henceforth the CDβGeo cells transiently treated with TGFβ for 14 days that sustain EMT after withdrawal of TGFβ will be referred to as persistently transdifferentiated (pTD) cells.

### The pTD cells have increased migration and invasion capability

**Figure 2:**
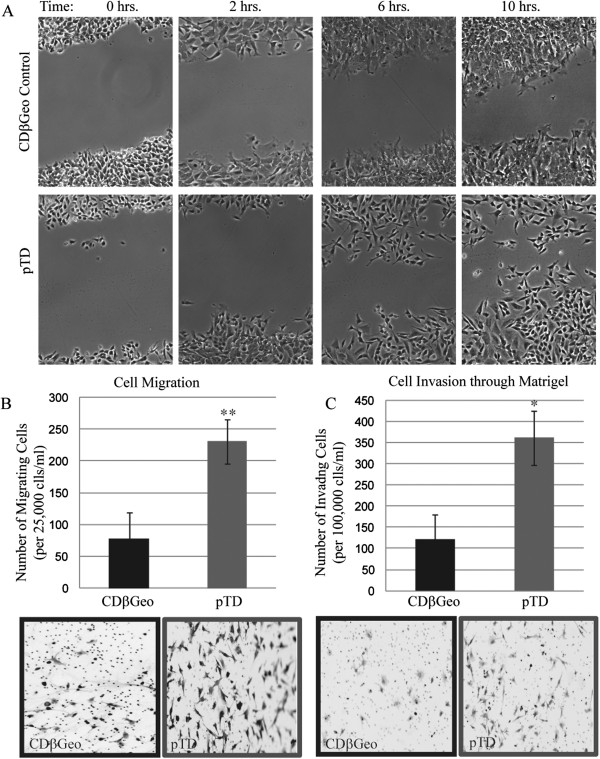
**pTD cells have increased migration and invasion capability. (A)** scratch assay demonstrates greater capability of pTD cells in wound closure. **(B)** pTD cells have greater migration capacity (**p < 0.01) and **(C)** greater invasion capability (*p < 0.05).

It is proposed that in cancer, EMT confers increased invasive ability to cancer cells. Therefore, we compared the migration and invasion capability of the CDβGeo parental cells and pTD cells. Scratch assays demonstrated that the CDβGeo control cells retain cellular attachment and slowly close the wound as an epithelial sheet (Figure 2A). In contrast, the pTD cells dissociate and migrate into the wound individually, and consequently, fill the gap more quickly and efficiently. Quantitative assessment of the migratory capability though culture inserts toward a chemoattractant (serum) shows that the pTD cells have enhanced migration capacity compared to CDβGeo control cells (p < 0.05; Figure 2B) and a 3-fold greater invasive capability through matrigel coated membranes (p < 0.01; Figure 2C).

### The transplanted pTD cells are more tumorigenic *in vivo* than parental CDβGeo cells

**Figure 3:**
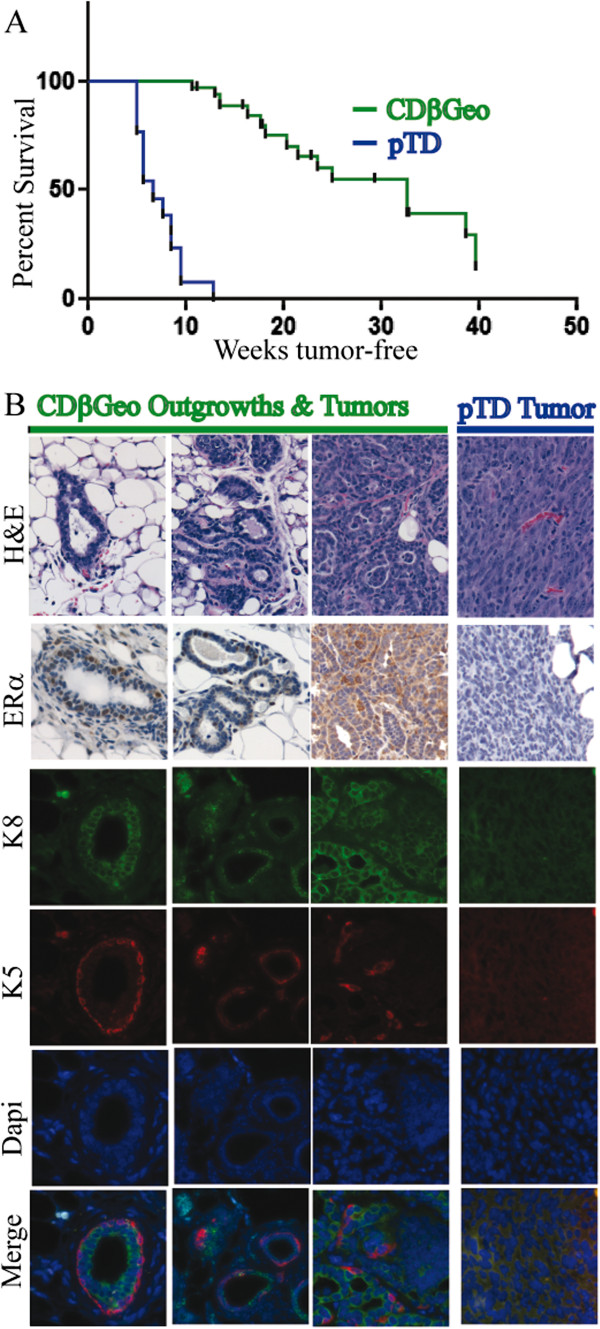
**Transplanted pTD cells are more tumorigenic *in vivo *than parental CDβGeo cells. (A)** Survival curve for mammary tumour incidence in weeks. pTD cell transplants are significantly more tumorigenic than CDβGeo cells (p < 0.01). **(B)** Immunohistochemistry for CDβGeo normal-like ductal outgrowth (first column), hyperplastic outgrowth (second column), acinar adinocarcinoma (third column) and pTD tumours (fourth column): hematoxylin and eosin stain (first row); ERα expression (second row); immunofluorescence for K8 (third row); K5 (fourth row); Dapi (fifth row) and merge (sixth row). Scale bar = 20 μm.

To compare their tumorgenicity, 50 000 CDβGeo parental cells and 50 000 pTD cells were transplanted into contralateral cleared fat pads of thirteen 3-week old BALB/c mice. Large tumours developed so rapidly from the pTD transplants (100%; mean latency 6.7 weeks) that the study had to be concluded by 13 weeks and did not allow for adequate assessment of the CDβGeo parental cells. Therefore, 50 000 CDβGeo cells were transplanted into both cleared fat pads to allow assessment of tumorgenicity of the parental cells (Figure 3A). CDβGeo cells produce outgrowths with normal ducts as well as alveolar hyperplasia. The outgrowths of CDβGeo cells are pre-neoplastic, producing mammary tumours in less than 43% of transplants with a longer mean latency (32.7 weeks) compared to pTD cells (p < 0.001). These results demonstrate that transient TGFβ-treatment transforms mammary epithelial cells making them more tumorigenic *in vivo*.

The characteristics of outgrowths and tumours from the CDβGeo and pTD cells were determined using immunohistochemistry for estrogen receptor alpha (ERα) and K8 to define luminal cells and K5 to identify basal epithelia (Figure 3B). CDβGeo outgrowths ranged from normal glandular, ductal hyperplasia, ductal adenocarcinoma with acinar morphology and, in some cases, solid de-differentiated tumours. Most of the normal appearing CDβGeo ductal outgrowths expressed ERα, K8 and K5 appropriately (Figure 3, left column). 56% of the CDβGeo tumours examined were ERα positive, but there was no correlation between the expression of ERα and tumour development as some ductal structures were ERα negative and some solid tumours were ERα positive. As outgrowths progressed from normal-like, to ductal hyperplasia and ductal adenocarcinoma, expression of both K8 and K5 were progressively lost. In contrast, the pTD outgrowths did not have any normal ductal architecture. All the pTD tumours were solid sheets of de-differentiated spindle like cells (Figure 3B, right column). The pTD tumours were locally invasive into muscle tissue and into the body cavity. In the tumours that were positive for ERα (24%), expression was weak. Likewise, expression of K8 and K5 were weak or absent. We conclude that transient *in vitro* TGFβ-treatment advances the tumorgenicity of the cells such that the pTD transplants produce more aggressive solid de-differentiated tumours.

### Characterization of gene expression changes in the pTD cells

We also examined the transcriptional profiles of genes differentially regulated relative to the CDβGeo parental cells to further characterize the pTD cells. Analysis with DAVID Bioinformatics Resources [36,37] using a subset of 482 up-regulated and 563 down-regulated DAVID IDs (>2-fold change; p < 0.05), identified significant increases in ECM-receptor interactions and focal adhesion in the pTD cells (Additional file 2: Table S1). The pTD cells also demonstrated decreases in cell cycle, DNA replication, p53 signalling and tight junction pathways. The normal mammary duct is comprised of luminal epithelial cells, basal cells and a small population of stem cells. Profiles of genes defining luminal epithelial or basal cells are decreased in the pTD cells relative to the CDβGeo cells (Additional file 3: Figure S2A & B). Many luminal epithelial junction proteins including the claudins, junction plakoglobin (JUP), epithelial cell adhesion molecule (EpCAM) and the epithelial keratins are down-regulated in the pTD cells relative to the CDβGeo cells. Likewise, basal keratins, smooth muscle actin and actin interacting proteins are also down-regulated in the pTD cells. This apparent de-differentiation of cultured cells by TGFβ-treatment agrees with the loss of differentiation markers in the pTD tumours. Genes in a profile that defines stem cells are also down-regulated (Additional file 3: Figure S2C). There are no increases in the surface markers used to sort stem cells (CD44, CD49f, or CD29) and no increase in stem cell associated transcription factors (Hey1, Nanog, Pou5F1/Oct4 or Sox9). However, Snai2, up-regulated during EMT and in stem cells, is increased in the pTD cells (Additional file 3: Figure S2D). Profiles defining genes regulated during EMT are persistently altered in the pTD cells, including, 2-fold up-regulation of fibronectin, N-cadherin, vimentin, Snai1, Twist, and many matrix metalloproteinases (MMP), along with 2-fold down-regulation of E-cadherin (Cdh1), bone morphogenic proteins (BMPs), and secreted frizzled-related protein (Sfrp1) (p < 0.05). There are also significant changes in the expression of components of the ECM. The pTD cells also have increased expression of growth factor promotion genes including growth factors, cytokines and growth factor receptors, while tumour suppressors are down-regulated. We conclude that transient TGFβ-treatment transforms CDβGeo cells such that they are de-differentiated and persistently transdifferentiated with increased expression of EMT markers, changes in ECM components and increased sensitivity to tumour promotion.

### Persistently transdifferentiated pTD cells do not exhibit an increase in the stem cell pool

**Figure 4:**
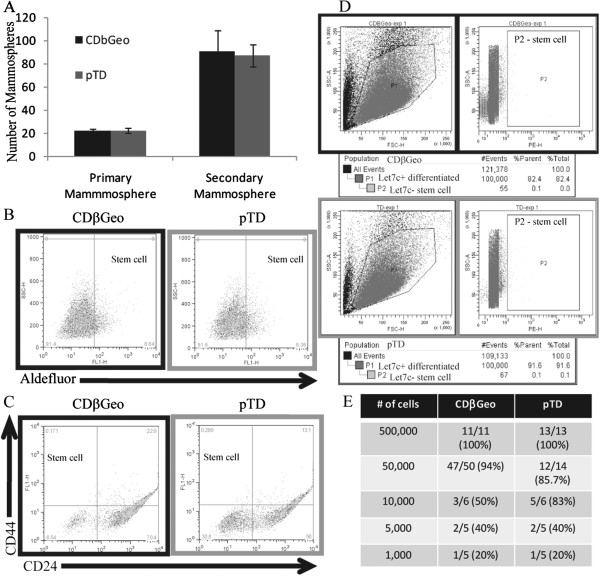
**Persistently transdifferentiated pTD cells do not have an increase in the stem cell pool. (A)** Initiation of primary or secondary mammospheres are not different between CDβGeo cells and pTD cells. **(B)** FACS for Aldefluor. **(C)** FACS for CD44 CD24. **(D)** FACS for Let7c sensor. **(E)** Limiting dilution for CDβGeo and pTD cells.

Previous reports suggest that the population of stem cells is increased during the implementation of a transient EMT program [20,25,26]. Because stem cell profiles and EMT profiles overlap, and because the pTD cells demonstrate persistent EMT-mediated changes in gene expression without increases in select stem cell genes, we compared stem cell ratios relative to the CDβGeo parental cells using three distinct assays. During the TGFβ exposure period, the mammosphere forming capability is transiently increased (Additional file 4: Figure S3), but although EMT is persistent 14 days after withdrawal of TGFβ, on day 28 there was no increase in primary or secondary mammosphere formation (Figure 4A). FACs analysis also shows no difference in aldefluor positive cells or changes in the CD44^High^ CD24^Low^ population (Figure 4 B&C). The let-7c sensor assay, which utilizes the fact that stem cells express very low let7c microRNA [38], also demonstrates no differences in the proportion of stem cells between the CDβGeo and pTD cells (Figure 4D). A limiting dilution series also shows that the CDβGeo cells and the pTD cells have similar capacity to repopulate in the mammary gland (Figure 4E). Specifically, partial growth (<50% of fat pad filled) occurred equally between the two cell types when 5000 (2/5) or 1000 (1/5) cells were transplanted. Regardless of the number of cells transplanted, the successful pTD outgrowths always produced solid tumours, even when examined as early as three weeks after transplantation. We find no evidence that there is an increase in the stem cell population in the pTD cells.

### Expression of snail, Zeb2 and Sfrp1 are altered in transdifferentiated cells

**Figure 5:**
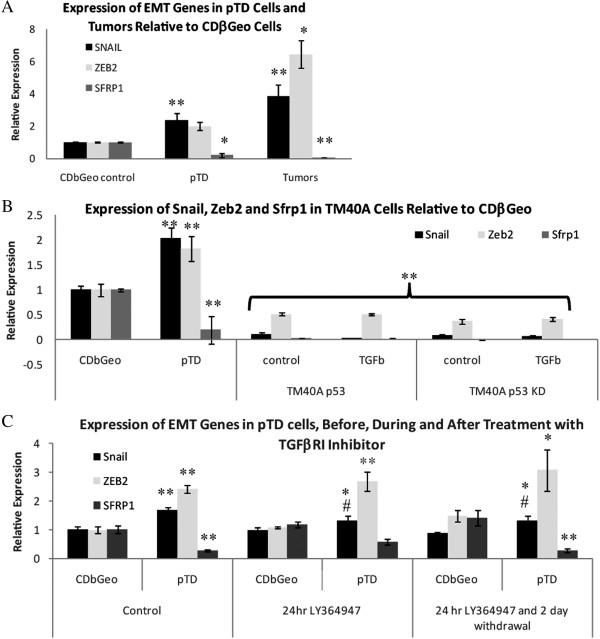
**Expression of Snail**, **Zeb2 and Sfrp1 are altered in transdifferentiated cells. (A)** QPCR expression of Snail, Zeb2 and Sfrp1 in pTD cells and tumours relative to CDβGeo. **(B)** QPCR expression of Snail, Zeb2 and Sfrp1 relative to CDβGeo cells in TM40A cells with wild type p53 or p53 knock-down maintained in control media or TGFβ media. *p < 0.05; **p < 0.01. **(C)** Changes in expression of Snail, Zeb2 and Sfrp1 relative to CDβGeo cells (*p < 0.05, **p < 0.01) and relative to pTD cells (#p < 0.05, ##p < 0.01) after 24 hour treatment with TGFβ inhibitor LY364947 and 48 hours after withdrawal of LY364947 demonstrate that autocrine production of TGFβ2 may support persistent EMT.

Up-regulation of Snail and Zeb2, along with repressed expression of Sfrp1, are features of EMT which contribute to mammary tumours [31,39]. As expected, quantitative RT-PCR demonstrates elevated expression of both Snail and Zeb2 in the pTD cells (2-fold) and tumours (>4-fold) relative to the CDβGeo cells along with suppression of Sfrp1 (Figure 5A). The expression changes in these three genes serve as an indication of EMT.

Persistent EMT does not occur in mammary epithelial cells unless they have already been transformed by an oncogenic mutation; specifically activated Ras [21,25]. As CDβGeo cells are p53-deficient [34] we tested the effect of p53-deficiency in rendering cells permissive to EMT. The TM40A mammary epithelial cell line is also derived from BALB/c mice, but retains wildtype p53 and are non-tumorigenic [38]. The TM40A cells do not undergo EMT in response to TGFβ-treatment, and cells expressing siRNA to knock down p53 (TM40A si-p53) also do not undergo EMT in response to TGFβ. Likewise, there are no differences in the expression of Snail, Zeb2 or Sfrp1 between TM40A control, TM40A TGFβ-treated, TM40A p53 si-control or TM40A si-p53 following TGFβ treatment (Figure 5B). We conclude that p53 deficiency does not contribute to sustained EMT and transdifferentiation in the CDβGeo cells by TGFβ.

In contrast to CDβGeo cells, basal levels of Snail and Zeb2 are significantly lower (p < 0.01) in the TM40A cells and were not altered by TGFβ. The TM40A cells are responsive to TGFβ as determined by CAGA-luciferase reporter assay (Additional file 5: Figure S4A). Likewise, another mouse mammary cell line, NMuMG, also has lower endogenous Snail expression relative to CDβGeo cells (Additional file 5: Figure S4B), and this cell line has been shown to be only capable of transient EMT in response to TGFβ [23]. These results suggest that the elevated endogenous levels of Snail and Zeb2 may render CDβGeo cells sensitive to transdifferentiation by TGFβ.

### Autocrine production of TGFβ2 contributes to persistent EMT

The expression of ligands in the transforming growth factor beta superfamily identified persistent up-regulation of TGFβ2 (p < 0.01) in pTD cells. Autocrine production of TGFβ ligand can support EMT and tumorigenesis [21,40,41]. To test if persistent EMT was maintained by an autocrine TGFβ positive feedback loop, we examined the expression of Snail, Zeb2 and Sfrp1 during and after treatment with the TGFβRI inhibitor LY364957 (10 μM). LY364957-treatment had no effect on the parental CDβGeo cells, neither at 24 hours after treatment nor subsequent to a 24 hour inhibitor treatment followed by a 48 hour withdrawal of inhibitor (Figure 5C). However, there is partial suppression of Snail expression in the pTD cells relative to pTD control (p < 0.05) after treatment with the inhibitor that is sustained after inhibitor withdrawal. Sfrp1 expression is also restored after 24 hour LY364947-treatment, but Sfrp1 rescue is not sustained after removal of the inhibitor. Inhibition of autocrine TGFβ signalling had no effect on Zeb2 expression in the pTD cells. These results demonstrate that in persistently transdifferentiated mouse mammary epithelial cells, a transient 24 hour block of autocrine TGFβ signalling can initiate a partial rescue of gene expression for Snail and Sfrp1, but not Zeb2.

## Discussion

CDβGeo transplants are considered premalignant because they form hyperplastic outgrowths, some of which progress to invasive tumours. Transient TGFβ-treatment of CDβGeo cells *in vitro* promotes EMT that is sustained after withdrawal (designated pTD cells) and transforms these mammary epithelial cells such that they become mesencymal-like and highly tumorigenic *in vivo*. The pTD cells, and the tumours that develop from them, are de-differentiated, having lost markers that define both luminal epithelial and myoepithelial cells. Interestingly, there is no comprehensive acquisition of stem cell markers, but rather decreased expression of several key stem cell markers including CD44, CD49f, CD29 and Sox9, with no change in the expression of Nanog or Pou5f1 (Oct4). This is consistent with Nguyen *et al*., who demonstrate that induction of TGFβ only accelerates tumorigenesis, and that radiation-induced notch signalling is required for expansion of mammary stem cells [42]. Although EMT has been reported to increase the population of cells with stem-like characteristics [20,25,43], TGFβ-induced persistent EMT in the CDβGeo cells was not accompanied by increases in the stem cell pool. Although CDβGeo cells clearly have mammary progenitors [33] the mammosphere forming capacity and transplant capability is similar to primary mouse mammary epithelial cells [44]. Similar to other reports [45,46], these cells do show enrichment of the stem cell pool during TGFβ-treatment *in vitro* (Additional file 4: Figure S3), but enrichment is transient, and the equilibrium in cell populations is restored upon subsequent passages and may not be essential for tumours.

The cancer stem cell theory proposes that only a small subset of cells, the tumour initiating cells, can seed a new tumour or a metastasis [47]. Therefore, there is great interest in identifying cancer stem cells in order to identify pathways and targets to reduce the metastatic potential of cancer. However, the defining line between EMT, mesenchymal cells, cancer stem cells and bulk tumour cells is indistinct [48,49] with substantial overlap among makers of EMT and profiles to define stem cells [29,30]. These signatures also align with human claudin-low and metaplastic breast cancers [41], although clearly these are not the only tumours with metastatic potential. The CDβGeo model identifies changes in ECM, MMPs, and transcription factors such as Snai1, Snai2, and Zeb2 as indicative of EMT. Because our model represents EMT without changes in the stem cell population, it suggests that ITGA6, DUSP6, Sox9, and KLF4 are valid markers for stem cells as suggested by Gupta et al. [45]. Because pTD cells demonstrate persistent EMT without increases in the stem cell pool, this model can be used to separate markers for EMT and consequently refine signatures that define tumour initiating cells.

Previous work has demonstrated that transdifferentiation of mammary epithelium in response to TGFβ-treatment is transient [19,24] and that sustained transdifferentiation and tumorigenesis *in vivo* only occurs with sustained TGFβ exposure or transformation with v-Ha-Ras oncogene [19]. Deletion of p53 also promotes EMT by releasing the repression of Zeb1, Zeb2 and BMI1 [50]. However, our experiments with TM40A cells show that blocking p53 is not sufficient for TGFβ-mediated EMT. Additionally, even though the CDβGeo cells are p53-deficient, cell growth was repressed by TGFβ. This agrees with other reports that TGFβ-mediated cell cycle arrest is p53-independent [51] and that p63/p73 may compensate in TGFβ-mediated pathways [52], including possibly those that promote EMT.

Persistent EMT has also been shown to be dependent on sustained TGFβ exposure through an autocrine positive loop [21,32,40,53]. The pTD cells have elevated TGFβ2 and there is partial rescue, with decreased expression of Snail and increased expression of Sfrp1, when the pTD cells are treated with the TGFβRI inhibitor LY364947. While higher doses of the TGFβRI inhibitor or a longer course of treatment may achieve a more robust rescue, the transcriptional profiles suggest that the transformed pTD cells have undergone epigenetic modifications, affecting multiple pathways, such that targeting TGFβ-pathways alone will not be effective. With extended expansion in culture (>5-10 additional passages), the pTD cells gradually regain a cobblestone epithelial morphology *in vitro*. This partial MET *in vitro* may be due to the dilution, during sequential passaging, of TGFβ2 and other factors that support the mesenchymal phenotype. EMT and acquisition of mesenchymal properties are necessary for some metastatic processes including intravasation, transport in circulation and extravasation. Dilution of mesenchymal supporting factors during dissemination may explain the paradox of why secondary tumours often exhibit an epithelial phenotype rather than a mesenchymal phenotype [54].

## Conclusions

Characteristics defining EMT and cancer stem cells are often synonymous. The CDβGeo model reveals that EMT is a separable state from stem cells; facilitating distinction to reveal targets important for the prevention and treatment of breast cancer metastasis. While our model reveals that the persistent EMT phenotype of the pTD cells are maintained by autocrine production of TGFβ2, targeting a single pathway (via TGFβ-inhibition) is not sufficient, illustrating the necessity of therapeutics targeting multiple pathways. Drugs targeting chromatin and epigenetic pathways offer a potentially valuable mechanism to silence EMT regulated genes and reverse oncogenic EMT.

## Methods

### Mice

All animals were bred and maintained in accordance with procedures approved by the Institutional Animal Care and Use Committee. 4th inguinal mammary fat pads were cleared as described [55] in female BALB/cMed recipient mice. CDβGeo and pTD cells (50 000 cells/10 μl) were injected with a Hamilton syringe and 30-guage needle into contra-lateral glands of thirteen hosts for tumour studies and were monitored for 13 weeks. Twelve additional mice received CDβGeo cells only in both glands and were monitored for 40 weeks. Cell and number of recipients for the limiting dilution experiments are described in Figure 4E. Glands for limiting dilution were processed for whole mounts as described [56] at 5 weeks to ascertain outgrowth potential.

### Cell culture and retroviral infection

CDβGeo cells were maintained in DMEM:F12 media (Sigma) supplemented with 2% adult bovine serum (Gibco), 10 μg/ml insulin (Sigma), 5 ng/ml mouse Epidermal Growth Factor (mEGF) and 100 U/ml Pen/Strep. pTD cells were generated by treating CDβGeo cells with 5 ng/ml TGFβ1 (R&D Systems Inc.) for 14 days during which control and treated cells were passaged 5 times to a similar density (1.2×10^6^ cells/100 mm plate every 3 days). Cell number and percent growth inhibition was determined with Vi-Cell cell viability analyzer (Beckman Coulter). Following the treatment period, the pTD and control cells were passaged (5 times) in maintenance media for an additional 14 days. (Figure 1A). TM40A-si-control and TM40A-si-p53 cells were generated and maintained as described previously [38] and treated with TGFβ or control solvent as described above.

### Flow cytometry

Fluorescence-Activated Cell Sorting (FACS) data were collected using LSRII (Becton Dickinson). A total of 100 000 events were collected and analyzed using DB FACSDiva software (Becton Dickinson).

### Immunocytochemistry, immunofluroescence and western blots

For cell culture, cells were grown to 100% confluency on laminin coated (2 μg/cm^2^) Lab-TekII glass chamber slides (154526, Nunc, Denmark). Cells were fixed with 2% paraformaldehyde, permeabilized with Karsenti’s Buffer (80 mM PIPES, 5 mM EGTA, 1 MM MgSO_4_, 0.5% Triton X-100), blocked in Protein Block (×0909; Dako) 20 minutes and incubated sequentially with primary antibody for 1 hour followed by secondary antibody for 1 hour.

CDβGeo and pTD outgrowth sections were deparaffinized and rehydrated prior to antigen retrieval in 10 mM citrate buffer for 20 minutes at 100°C. Primary antibodies for K5, K8 or ERα (MC-20) were used. Hematoxylin was used as a counterstain for ERα, while DAPI was used for immunofluorescence. All images were captured using a Nikon Eclipse TE2000-U and Metaview™ software (Universal Imaging Corporation). The Allred scoring system was used to determine ERα expression.

Cells were lysed with RIPA buffer (50 mM Tris, 150 mM NaCl, 1% Triton X-100, 0.1% SDS, 1% NaDeoxycholate [pH 7.4] supplemented with protease inhibitors (1 mM phenylmethylsulfonyl fluoride, 10 μg/ml pepstatin A, 10 μg/ml aprotinin, and 5 μg/ml leupeptin). Protein lysates were resolved by SDS-polyacrylamide gel electrophoresis (PAGE) and transferred onto Polyvinylidene Fluoride membrane (Millipore). Non-specific binding was blocked with PBS containing 0.2% Tween 20 and 5% nonfat dry milk, and blots were incubated 1 hour with primary antibody followed by incubation with horseradish peroxidase-conjugated secondary antibody, developed using enhanced chemiluminescence solution and visualized in G-Box imaging system (Syngene). Antibodies used are listed in Table 1.

**Table 1:** Antibodies

**Antigen**	**Cat. #**	**Source**	**Dilution**	**Application**
E-cadherin	610181	BD Biosciences	1:2000	WB
			1:50	IF
N-cadherin	610910	BD Biosciences	1:2000	WB
β-actin	4967	Cell Signaling	1:4000	WB
ZO-1	339100	Invitrogen	1:100	IF
K5	PRB-160	Covance	1:100	IF
K8	GP11	Progen Biotechnik	1:50	IF
ER (MC20)	SC545	Santa Cruz	1:200	IHC
AlexaFluor 568 conjugated phalloidin	A12380	Invitrogen	1:40	IF against actin

### Luciferase assay

CDβGeo, NMuMG and TM40A cells were transfected with 4 μg CAGA-luciferase plasmid and 0.05 μg Renilla(pRL-CMV) plasmid using Lipofectamine 2000 (Invitrogen). Luciferase assay was performed using Dual Luciferase Reporter Assay (Promega) and a 20/20^n^ Luminomer (Turner Biosystems).

### Mammosphere culture

CDβGeo cells and pTD cells were seeded at a density of 20 000 viable cells/ml in ultra low attachment dishes (3473, Corning Life Sciences) as described [38]. After collecting primary mammospheres with gentle centrifugation at 800 rpm for 5 minutes, cells were dissociated with 1 ml 0.05% trypsin-EDTA for 5–8 minutes and single cells were obtained by filtering cell suspension through a 40 μm cell strainer. Cells for secondary mammospheres were seeded at a density of 1000 viable cells/ml. Primary and secondary mammospheres were quantified by counting spheres >200 μm.

### Migration and invasion assays

For the scratch assay, CDβGeo and pTD cells were grown to 80% confluence. The wound was generated across the plate with a pipette tip. Images were captured every 2 hours for 12 hours with a Nikon Eclipse TE2000-U and Metaview™ software (Universal Imaging Corporation). For chamber migration assays, CDβGeo and pTD cells were seeded in serum free media into either BD BioCoat control chambers (25 000 cells/ml) or Matrigel™ invasion chambers (100 000 cells/ml) (BD Bio-sciences). Media containing 10% FBS was used as an attractant. After 22 hours incubation, cells were fixed for 10 minutes in 10% formalin, stained with Crystal Violet and membranes were mounted on microscope slides. Images were captured with an Olympic BX41 light microscope using SPOTSOFTWARE (Diagnostic Instruments, Inc.) and quantified using Image J.

### RNA isolation for quantitative RT-PCR and microarray

Total RNA was extracted using Trizol reagent according to manufacturer’s instructions (Invitrogen) and cleaned up with Qiagen RNeasy (Qiagen). Relative levels of mRNA were determined by quantitative real-time PCR. The assays were performed using the 1-step Brilliant® SYBR® Green QRT-PCR Master Mix Kit (Stratagene); primer sequences are listed in Table 2 and described previously [31].

**Table 2:** Primer sequences

**mRNA**	**Forward**	**Reverse**
**Snail**	gtctgcacgacctgtggaa	caggagaatggcttctcacc
**Zeb2**	ccagaggaaacaaggatttca	aggcctgacatgtagtcttgtg
**Sfrp1**	gctgctcaacaagaactgccacat	acatttgagcatctcgggccagta
**Pgk1**	tgactttggacaagctggacgtga	tgagttcagcagcaactggctcta

RNA samples (10 μg) were processed by the UCLA Microarray Core Facility and hybridized to the Affymetrix Mouse Genome 430 2.0 array (Affymetrix). The quality of the RNA and labelled cRNA were determined using the RNA 6000 Nano-LabChips (Agilent Technologies). Array quality, background correction and data normalization of gene expression data were computed directly from the Affymetrix. CEL files using the Bioconductor packages for R [57] implementation of affyPLM and Robust Multichip Average [58-60]. Differential expression of genes was determined using TM4 software [61]. Pair wise comparisons of each treatment relative to the vehicle-treated group was used to identify statistically differentially expressed probes (Welch’s t-test; P < 0.05 calculated from 1000 permutations of the expression data for each gene). DAVID [36,37] was used to investigate differences in signalling pathways. The genes for DAVID analysis were selected for >2-fold differences relative to control. The gene lists identifying Luminal, Basal, Stem Cells, EMT, ECM and Growth Factor Signalling were selected from those published previously [29,30,62-64].

### Statistical analysis

The tumour-free survival was analyzed using survival distribution with censoring in GraphPad Prism. The differences in tumour incidences were determined by the chi-square test and differences in expression in pTD cells relative to CDβGeo control were determined using the two-tailed Student’s t test. A p-value <0.05 was considered statistically significant.

## Abbreviations

TGFβ: Transforming growth factor beta; EMT: Epithelial to mesenchymal transition; MET: Mesenchymal to epithelial transition; PABC: Pregnancy associated breast cancer; pTD: persistently transdifferentiated CDβGeo cells; K8: Cytokeratin 8; K5: Cytokeratin 5; ERα: Estrogen receptor alpha; MMP: Matrix metalloproteinase; BMP: Bone morphogenic protein

## Competing interests

The authors declare that they have no competing interests.

## Authors’ contributions

KAD contributed to design of experiments, *in vivo* experiments, scratch and migration and invasion assays, immunofluorescence, microscope images, heat maps and statistical analysis. JHS contributed to initial concepts and protocol development for cell culture and treatment, and the *in vivo* experiments. DJK contributed to western blotting and mammosphere assays. ALR contributed to *in vivo* experiments and QRT-PCR. LT contributed to *in vivo* experiments and Let7 sensor assay. JD and ALB contributed to the FACS analysis for aldefluor and CD44 CD24. GMC helped with analysis of outgrowths and tumour classification. MJH normalized microarray data and contributed to profile analysis. TC contributed to immunofluorescence. KJG and SSS produced QRT-PCR for Zeb2 and Sfrp1. DJJ contributed to concepts, design and manuscript preparation. All authors read and approved the final manuscript.

## Supplementary Material

Additional file 1: Figure S1 **(A)** TGFβ-treatment restricts the growth of CDβGeo cells (percent relative to mean control) and **(B)** Quantification of immunofluorescence demonstrates TGFβ-treatment increases the K5 positive cell population (p < 0.05). Click here for file

Additional file 2: Table S1 DAVID Bioinformatics Database Annotation Summaries for pathways (KEGG_Pathway). Click here for file

Additional file 3: Figure S2 Characterization of gene expression changes in the pTD cells. Gene profiles in the pTD cells relative to the CDβGeo parental cells that characterize specific mammary cell types show decreased expression (green) for **(A)** luminal epithelium, **(B)** basal epithelium, and **(C)** stem cells. **(D)** An EMT gene profile shows increased expression (red) for genes up-regulated during EMT (CAV1 to ZEB2) and decreased expression (green) for genes down-regulated during EMT (BMP to WNT7B). **(E)** Expression changes in ECM genes. **(F)** Many growth factors, cytokines and receptors are up-regulated (red) while tumour suppressors are down-regulated (green). Log2 expression changes in CDβGeo and pTD cells relative to mean CDβGeo are shown in A-C. Mean log2 expression changes in the pTD cells relative to mean CDβGeo are shown in E-G. Click here for file

Additional file 4: Figure S3 Mammosphere forming capability is increased by TGFβ during (day 12) TGFβ-treatment (p < 0.01). Click here for file

Additional file 5: Figure S4 **(A)** Fold increase in TGFβ-induced luciferase in mouse mammary cell lines. **(B)** Endogenous Snail expression is lower in cell lines that fail to undergo persistent EMT in response to TGFβ. (Also shown for comparison expression of Pgk1 normaliser with normalized and non-normalized Snail expression; p < 0.01). Click here for file
