# Supplementary figures and images for: Oncogenic transformation of mammary epithelial cells by transforming growth factor beta independent of mammary stem cell regulation
Source: Cancer Cell Int. 2013 Jul 25;13:74. doi: 10.1186/1475-2867-13-74 (PMC3733955; doi:10.1186/1475-2867-13-74)

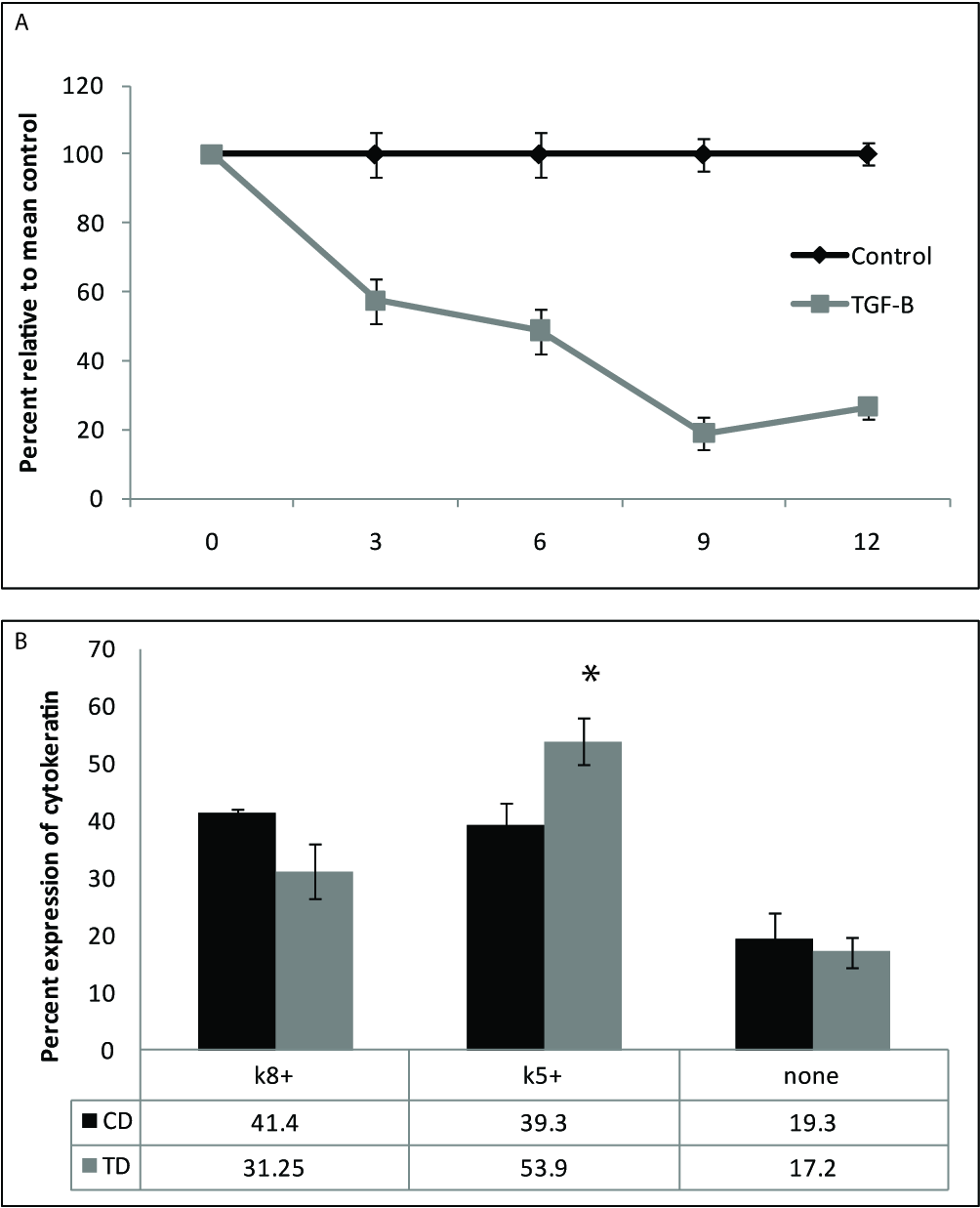

Supplement: Additional file 1: Figure S1 — (A) TGFβ-treatment restricts the growth of CDβGeo cells (percent relative to mean control) and (B) Quantification of immunofluorescence demonstrates TGFβ-treatment increases the K5 positive cell population (p < 0.05). [file 1475-2867-13-74-S1.tiff]

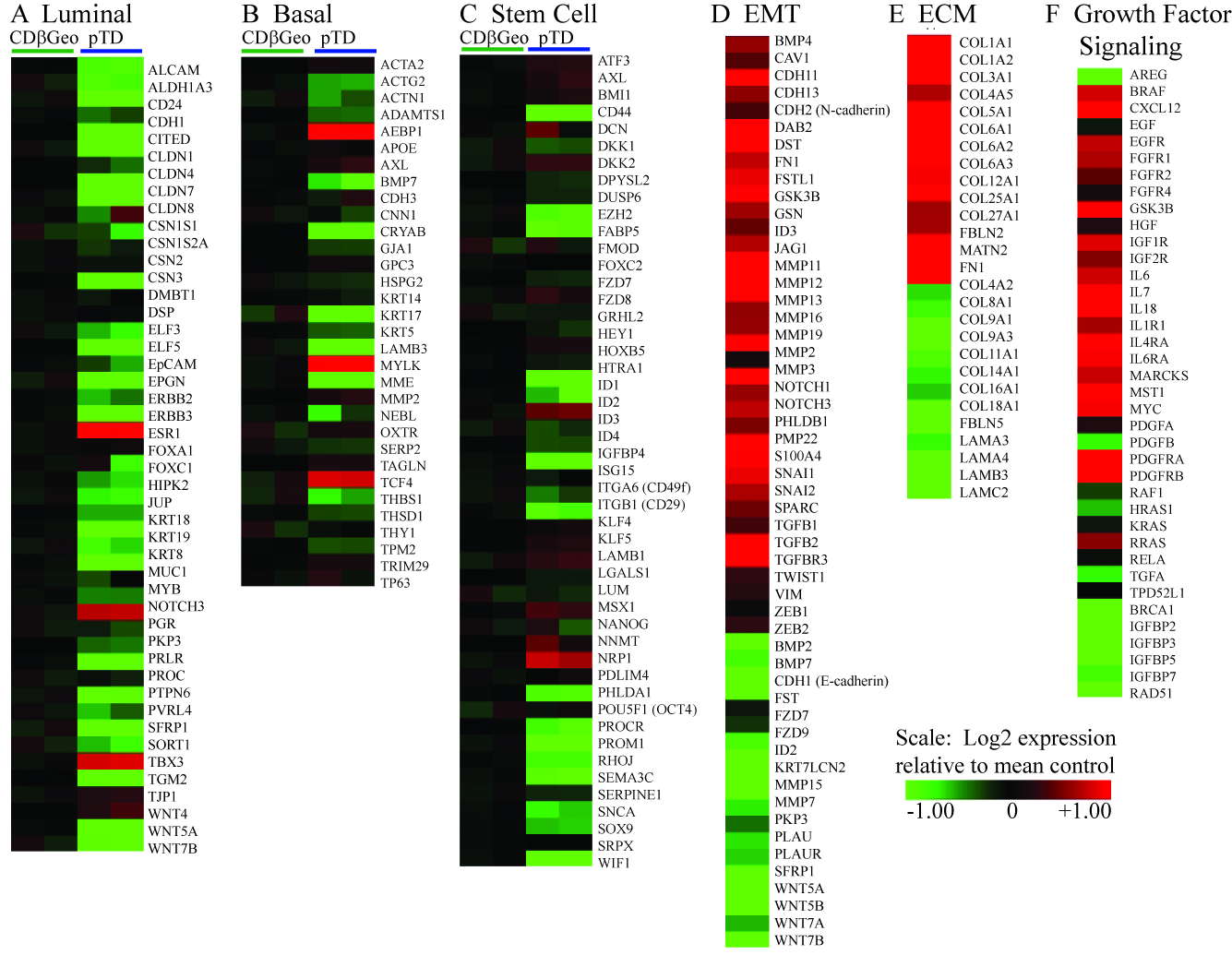

Supplement: Additional file 3: Figure S2 — Characterization of gene expression changes in the pTD cells. Gene profiles in the pTD cells relative to the CDβGeo parental cells that characterize specific mammary cell types show decreased expression (green) for (A) luminal epithelium, (B) basal epithelium, and (C) stem cells. (D) An EMT gene profile shows increased expression (red) for genes up-regulated during EMT (CAV1 to ZEB2) and decreased expression (green) for genes down-regulated during EMT (BMP to WNT7B). (E) Expression changes in ECM genes. (F) Many growth factors, cytokines and receptors are up-regulated (red) while tumour suppressors are down-regulated (green). Log2 expression changes in CDβGeo and pTD cells relative to mean CDβGeo are shown in A-C. Mean log2 expression changes in the pTD cells relative to mean CDβGeo are shown in E-G. [file 1475-2867-13-74-S3.tiff]

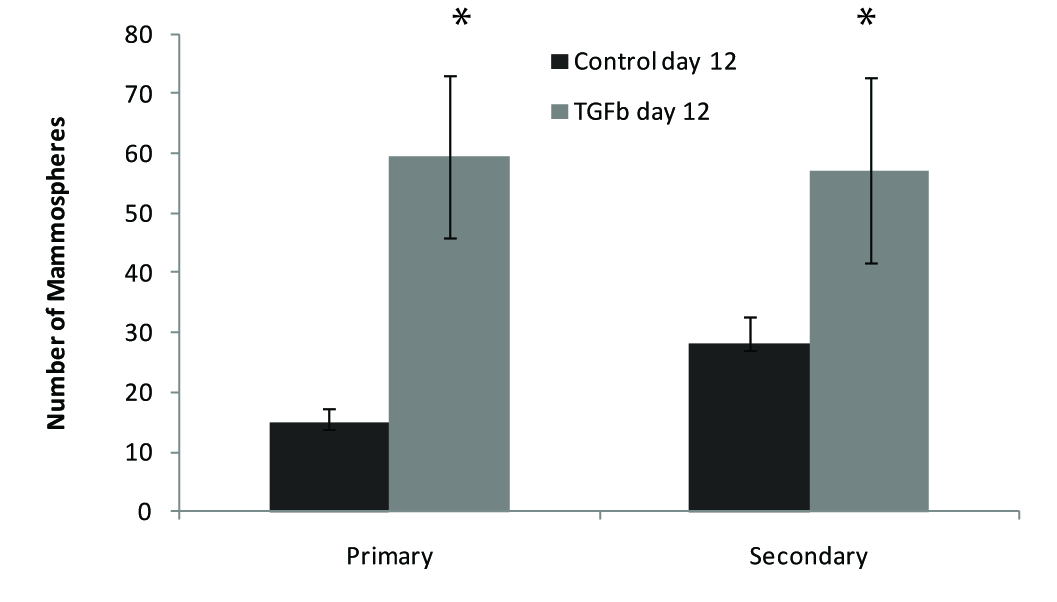

Supplement: Additional file 4: Figure S3 — Mammosphere forming capability is increased by TGFβ during (day 12) TGFβ-treatment (p < 0.01). [file 1475-2867-13-74-S4.tiff]

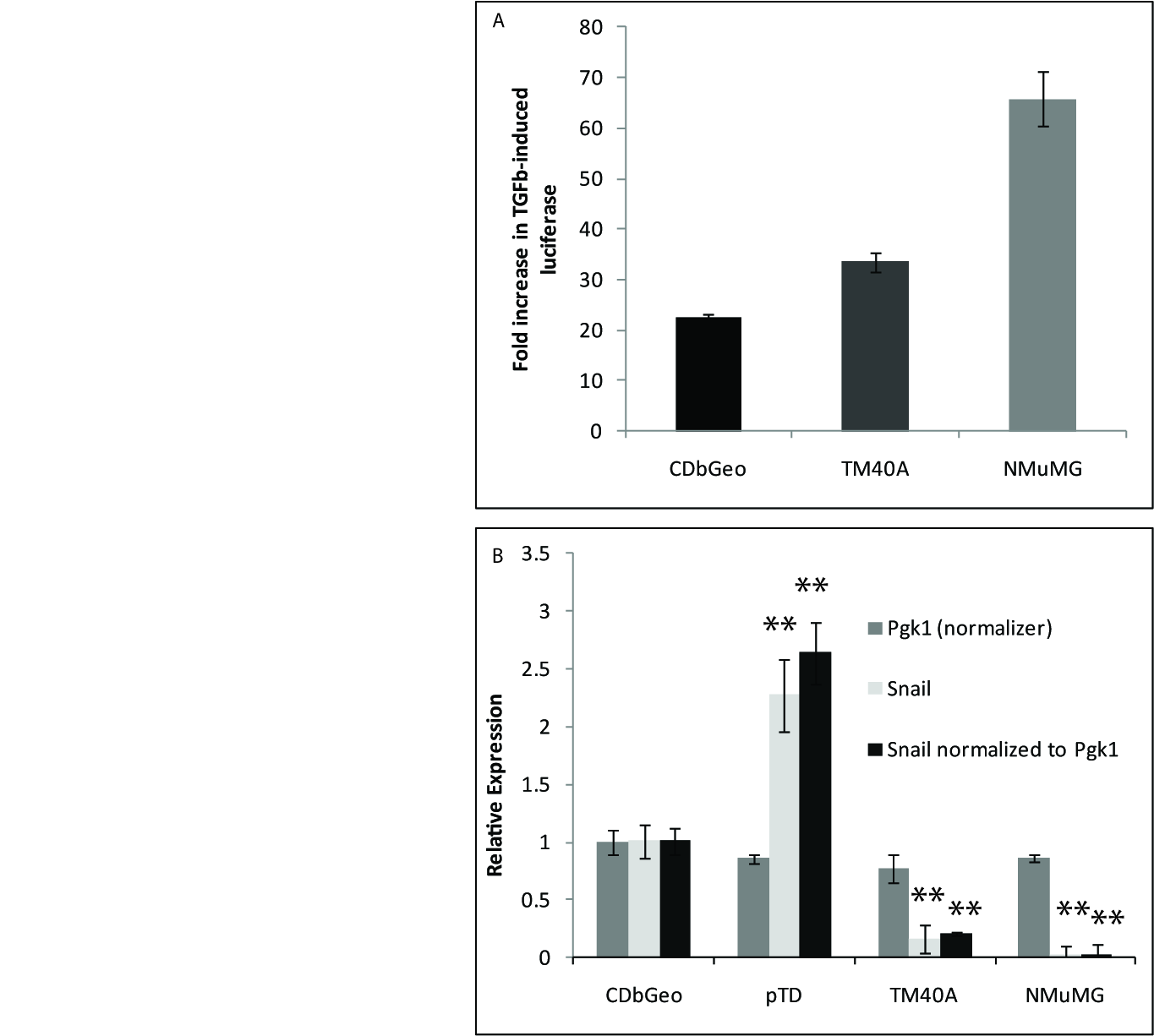

Supplement: Additional file 5: Figure S4 — (A) Fold increase in TGFβ-induced luciferase in mouse mammary cell lines. (B) Endogenous Snail expression is lower in cell lines that fail to undergo persistent EMT in response to TGFβ. (Also shown for comparison expression of Pgk1 normaliser with normalized and non-normalized Snail expression; p < 0.01). [file 1475-2867-13-74-S5.tiff]
