# Supplementary material for: Oncogenic transformation of mammary epithelial cells by transforming growth factor beta independent of mammary stem cell regulation
Source: Cancer Cell Int. 2013 Jul 25;13:74. doi: 10.1186/1475-2867-13-74 (PMC3733955; doi:10.1186/1475-2867-13-74)
Supplement: Additional file 2: Table S1 — DAVID Bioinformatics Database Annotation Summaries for pathways (KEGG_Pathway). [file 1475-2867-13-74-S2.docx]

DAVID Bioinformatics Database Annotation Summaries for pathways (KEGG_Pathway)

Identified from 482 up-regulated DAVID IDs

| Term | Gene count | p-value | Benjamini |
| --- | --- | --- | --- |
| ECM-receptor interaction | 12 | 2.9E-6 | 3.5E-4 |
| Metabolism of xenobiotics by cytochrome P450 | 10 | 1.9E-5 | 1.2E-3 |
| Focal adhesion | 16 | 5.0E-5 | 2.0E-3 |
| Lysosome | 11 | 4.2E-4 | 1.3E-2 |
| Drug metabolism | 8 | 1.7E-3 | 4.1E-2 |

Identified from 563 down-regulated DAVID IDs

| Term | Gene count | p-value | Benjamini |
| --- | --- | --- | --- |
| Cell cycle | 31 | 1.8E-17 | 2.4E-15 |
| DNA replication | 14 | 6.1E-11 | 4.0E-9 |
| p53 signaling pathway | 10 | 6.1E-4 | 1.6E-2 |
| Pyrimidine metabolism | 11 | 1.8E-3 | 3.0E-2 |
| Pathways in cancer | 23 | 1.9E-3 | 3.6E-2 |
| Tight junction | 13 | 2.6E-3 | 4.2E-2 |
